# Cardiovascular risk in persons at risk of developing rheumatoid arthritis

**DOI:** 10.1371/journal.pone.0237072

**Published:** 2020-08-03

**Authors:** Laurette van Boheemen, Marian H. van Beers-Tas, Johanna M. Kroese, Lotte A. van de Stadt, Dirkjan van Schaardenburg, Michael T. Nurmohamed

**Affiliations:** 1 Department of Rheumatology, Amsterdam Rheumatology and Immunology Center, Reade, Amsterdam, The Netherlands; 2 Departments of Orofacial Pain and Dysfunction, Periodontology, and Preventive Dentistry, Academic Center for Dentistry Amsterdam, University of Amsterdam and Vrije Universiteit Amsterdam, Amsterdam, The Netherlands; 3 Department of Rheumatology, Amsterdam University Medical Centers, AMC, Amsterdam, The Netherlands; 4 Department of Rheumatology, Amsterdam University Medical Centers, VUmc, Amsterdam, The Netherlands; University of Milano, ITALY

## Abstract

**Background:**

Rheumatoid arthritis (RA) is associated with an increased cardiovascular disease (CVD) risk which may start even before diagnosis. To explore this CVD risk prior to RA, we determined multiple risk factors and two 10-year clinical risk scores in a cohort of individuals at-risk of RA. We also analyzed associations with arthritis development and autoantibody status and compared a subset of at-risk individuals to an age and sex matched seronegative control group.

**Methods:**

In a cohort of 555 consecutive arthralgia patients positive for rheumatoid factor (RF) and / or anti-citrullinated protein antibody (ACPA) we retrospectively identified patients with preclinical arthritis (i.e. those who developed arthritis), and non-arthritis patients (those without arthritis development during maximum 5 years follow up). Demographics, CVD risk factors and the 10-year cardiovascular risk according to the SCORE and QRISK3 system were determined at baseline.

**Results:**

Preclinical arthritis patients (n = 188) had a higher heart rate (68 vs 63 bpm, p = 0.048) and lower cholesterol (5.2 mmol/l vs 5.5, p = 0.006), HDL (1.0 mmol/l vs 1.1, p0.003) and ApoB (0.85 g/l vs 0.91, p = 0.011) compared to non-arthritis patients (n = 367). Lipid levels were associated with ACPA status in both the preclinical arthritis and non-arthritis group. Ten-year CVD risk scores did not differ between preclinical arthritis and non-arthritis patients, in total, 7% (SCORE) and 8% (QRISK3) of seropositive arthralgia patients were classified as high risk. Seropositive at-risk patients (n = 71) had higher total cholesterol (5.4 vs 4.9, p<0.001), TC/HDL ratio (4.0 vs 3.0, p<0.001), triglycerides (1.4 vs 1.0, p = 0.001), ApoB (1.0 vs 0.9, p = 0.019) and 10-year risk scores (median SCORE 1.0 vs 0.0, p = 0.030 and median QRISK3 4.4 vs 3.1, p<0.001) compared to seronegative controls.

**Conclusion:**

Our results suggest that lipid changes commence prior to RA diagnosis and that ACPAs might play a role.

## Introduction

Rheumatoid arthritis (RA) is a systemic inflammatory autoimmune disease associated with an increased cardiovascular disease (CVD) risk that is already present at the time of diagnosis [1–6]. There is also some evidence of increased CVD risk prior to clinically manifest RA. In patients who later developed RA, unfavorable lipid profiles [7, 8] and an increased risk of coronary heart disease were found [9]. Moreover, antibodies to citrullinated proteins (ACPA) have been associated with coronary artery disease, even in the absence of auto-immune disease [10]. It is hypothesized that auto-immune mediated processes might already increase CVD risk in the preclinical phase of RA. The etiology of the increased risk might be an interplay of several determinants including traditional CVD risk factors, systemic low grade inflammation and autoimmunity to post-translational modification of proteins [11–13].

Exploring a comprehensive cardiovascular risk factor profile as well as an overall 10-year risk estimation of cardiovascular events in a large cohort of patients at-risk for RA enabled us to study the presence and extent of CVD risk prior to RA diagnosis. To calculate the 10-year CVD risk, the European Guidelines on Cardiovascular Disease prevention in clinical practice recommend using the SCORE (Systematic Coronary Risk Evaluation) in which the risk score is multiplied by 1.5 for RA patients [14]. Additionally, the QRISK system is widely used in clinical practice in RA patients since its algorithm includes RA as an independent risk factor [15]. The distribution of SCORE has previously been described in different populations of RA patients, where 14% to 20% of patients were classified as high or very high risk [16, 17]. A study using the QRISK3 score in RA patients, with a mean disease duration of 11.4 years, classified 30% of patients as high risk [18]. The distribution of SCORE and QRISK3 in a population at-risk for RA has thus far not been reported.

Information about the presence and extent of CVD risk prior to RA may help to better understand the etiology and causal relationships between RA and CVD. It may also help to determine if cardiovascular screening is necessary in a population at increased risk of RA. Therefore, our primary objective was to explore a wide range of CVD risk factors and the 10-year risk of cardiovascular events in a large cohort of seropositive arthralgia patients at increased risk of developing RA, and comparing persons who did and did not develop arthritis during follow up. Secondly, we analyzed associations between CVD risk and autoantibody status. Additionally, in a subset of persons at risk of RA, CVD risk was compared to seronegative controls.

## Patients and methods

### Study participants

Five-hundred fifty five consecutive patients from the Reade seropositive arthralgia cohort, included between August 2004 and May 2017, with a follow up of ≥ 12 months or until arthritis development entered this study [19]. All patients were at-risk of arthritis defined by arthralgia (i.e. suspected inflammatory joint pains) and positivity for at least one serologic marker: rheumatoid factor (RF; >5 kU/l) and / or ACPA (>10 kU/l). There was no history and no presence of clinically diagnosed arthritis at the time of inclusion in the cohort. We retrospectively identified patients with preclinical arthritis (those who developed arthritis during follow up), and non-arthritis patients (patients without arthritis development during maximum 5 years follow up).

For the additional analyses, RF and ACPA negative individuals without any rheumatic disease from the general population were recruited between January 2018 and August 2019. All seronegative participants were matched for sex and age (with a range of +/- 3 years) in a 1:1 ratio to seropositive arthralgia patients, of which those with the best match were selected.

### Measurements

At baseline, demographics, medical history, medication use and presence of comorbidities were collected. Blood parameters, including autoantibodies and lipid profile, were determined. Lipid profile contained total cholesterol (TC, mmol/L), high density lipoprotein (HDL, mmol/L), low density lipoprotein (LDL, mmol/L), TC/HDL ratio, triglycerides (mmol/L), apolipoprotein A1 (ApoA, g/L) and apolipoprotein B (ApoB, g/L). In a subset of patients, those included from August 2012 onwards (n = 211), blood pressure (BP; using an automatic BP monitor) and heart rate (in beats per minute (bpm) either from an automatic BP monitor or a 12 lead ECG) were measured according to the standard hospital procedures. A physician performed the physical examination to confirm absence of arthritis. Following baseline assessment, all patients were reassessed at 12-month intervals during 5 years with a focus on development of clinical arthritis. In case of arthritis development, an extra visit was scheduled.

SCORE and QRISK3 were calculated for persons of whom data were complete (i. e. arthralgia patients included after August 2012). Persons with a history of CVD, defined as a history of coronary heart disease (myocardial infarction, angina pectoris, percutaneous coronary intervention, coronary artery bypass surgery, cerebral vascular disease and peripheral arterial disease) were excluded for the SCORE and QRISK3 analyses. The SCORE system uses age, sex, systolic blood pressure (SBP), TC, HDL and smoking status to calculate the 10 year risk of cardiovascular events. A SCORE risk <1% was classified as low risk, between 1% and 5% as intermediate risk and ≥5% as high risk. In addition to these variables, the QRISK3 system uses ethnicity, BMI, family history of coronary heart disease in a first degree relative aged less than 60 years, diabetes (type 1 and 2), RA, SLE, atrial fibrillation, chronic kidney disease (stage 3, 4 or 5), migraine, severe mental illness, erectile dysfunction, and use of antihypertensive medication, atypical antipsychotics or corticosteroids. A QRISK3 risk ≥20% was classified as high risk. Additionally, the QRISK3 tool provides a relative risk compared to the score of a healthy person of the same age, sex and ethnicity.

In the seronegative control group, baseline characteristics, lipid profile, BP and heart rate (measured using an automatic BP monitor) were collected.

### Statistical methods

Descriptive statistics were used to describe baseline characteristics, CVD risk factors and the distribution of SCORE and QRISK3. Differences were assessed between preclinical arthritis patients and non-arthritis patients. Secondly, baseline differences were assessed between ACPA positive and negative at risk patients. Additionally, differences were assessed between at risk patients and seronegative controls.

For all analyses containing lipid data, persons using cholesterol lowering drugs were excluded. Persons using antihypertensive drugs were excluded from analyses using blood pressure and heart rate. Continuous, normally distributed data were presented as mean (standard deviation) and analyzed by t-tests. Non-normally distributed data were presented as median (interquartile range) and analyzed by Mann-Whitney U tests. Binary data were analyzed for differences between groups by chi-square test or Fisher’s exact test if applicable. Statistical analyses were performed using SPSS V24.0.

The study was approved by the Ethics Committee of Slotervaart Hospital and Reade, Amsterdam, The Netherlands, and written informed consent was obtained from all study participants.

## Results

### Cardiovascular risk in preclinical arthritis patients and non-arthritis patients

Of 555 arthralgia patients, 75% was female, the mean age was 49.5 years (SD 11.6), 68% was RF positive and 63% was ACPA positive (Table 1). Twenty individuals (3.6%) had a history of CVD (50% female, 55% ACPA positive). Median follow up was 36 months (IQR 13–60). One-hundred and eighty-eight individuals (34%) developed arthritis after a median of 11 months after inclusion of which 92% fulfilled the 2010 ACR/EULAR classification criteria for RA and of which 165 persons (88%) were ACPA positive. Preclinical arthritis patients had a higher heart rate (p = 0.048) and lower TC (p = 0.006), HDL (p = 0.003) and ApoB levels (p = 0.011) compared to non-arthritis patients. VAS pain (which may affect heart rate) showed no significant difference and was 35 (14.3–59.8) and 28 (8–51) respectively, p = 0.117.

**Table 1:** Baseline characteristics of all seropositive arthralgia patients, and preclinical arthritis and non-arthritis patients separately.

	Seropositive arthralgia	Preclinical arthritis	Non-arthritis	p-value*
	N = 555	N = 188	N = 367	
Age (mean ±SD)	49.5 (11.6)	49.0 (11.3)	49.7 (11.8)	0.521
Sex, n (% female)	415 (75)	139 (74)	276 (75)	0.745
RF positive, n (%)	376 (68)	122 (65)	254 (70)	0.243
ACPA positive, n (%)	348 (63)	165 (88)	183 (50)	<0.001
CRP (median, IQR)	2.00 (1.00–4.70)	2.32 (1.00–4.72)	2.00 (1.00–4.70)	0.070
Statin users, n (%)	48 (9)	19 (10)	29 (8)	0.382
Antihypertensive drug users, n (%)	83 (15)	27 (14)	56 (15)	0.779
History of CVD, n (%)	20 (4)	6 (3)	14 (4)	0.709
DM, n (%)	21 (4)	7 (4)	14 (4)	0.951
Current smoker, n (%)	157 (28)	56 (30)	101 (28)	0.562
SBP^1^ (mean ±SD)	128.46 (16.29)	132.23 (19.56)	127.50 (15.31)	0.187
Heart rate^1^ (mean ±SD)	64.20 (9.63)	67.60 (8.04)	63.34 (9.85)	0.048
BMI^2^ (mean ±SD)	26.07 (4.66)	25.90 (3.81)	26.12 (4.87)	0.802
TC^3^ (mean ±SD)	5.39 (1.10)	5.20 (1.00)	5.48 (1.13)	0.006
HDL^3^ (mean ±SD)	1.08 (0.43)	1.00 (0.42)	1.12 (0.42)	0.003
TC/HDL ratio^3^ (median, IQR)	5.2 (3.93–6.94)	5.48 (4.05–7.46)	5.05 (3.86–6.76)	0.088
LDL^3^ (mean ±SD)	3.60 (1.03)	3.53 (0.96)	3.64 (1.06)	0.265
Triglycerides^3^ (median ±IQR)	1.32 (0.95–1.88)	1.36 (0.98–1.78)	1.32 (0.93–1.93)	0.976
ApoA^3^ (mean ±SD)	1.66 (0.34)	1.62 (0.35)	1.68 (0.34)	0.065
ApoB^3^ (mean ±SD)	0.89 (0.25)	0.85 (0.23)	0.91 (0.25)	0.011
SCORE^4^ (median, IQR)	1.00 (0.00–2.00)	1.00 (0.00–2.00)	1.00 (0.00–2.00)	0.379
QRISK3^4^ score (median, IQR)	3.5 (1.58–9.73)	4.7 (1.7–10.4)	3.3 (1.3–9.7)	0.293
QRISK3 relative risk score^4^ (median, IQR)	1.3 (1.0–1.83)	1.2 (0.9–1.7)	1.4 (1–1.9)	0.312

* p-value for preclinical arthritis versus non-arthritis patients

^1^ Measured in a subset; people with antihypertensive medication use were excluded from analyses. Included: n = 128 (preclinical arthritis: 26, non-arthritis: 102).

^2^ Height and weight was measured in a subset. Included: n = 160 (preclinical arthritis: 34, non-arthritis: 126).

^3^ Subjects using cholesterol lowering medication were excluded from analyses. Included: n = 507 (preclinical arthritis: 169, non-arthritis: 338).

^4^ For SCORE and QRISK3 calculation, subjects with a history of CVD were excluded. Included: n = 150 (preclinical arthritis: 31, non-arthritis: 119).

ACPA: anti-citrullinated protein antibodies, ApoA: apolipoprotein A, ApoB: apolipoprotein B, BMI: body mass index, CRP: C-reactive protein, CVD: cardiovascular disease, DM: diabetes mellitus, HDL: high density lipoprotein, LDL: low density lipoprotein, RF: rheumatoid factor, SBP: systolic blood pressure, TC: total cholesterol, TC/HDL ratio: total cholesterol / high density lipoprotein ratio.

SCORE and QRISK3 were calculated in 150 patients. Compared to the remaining 405 patients, sex and age did not differ, but slightly less patients were ACPA positive (55% vs 65%, p = 0.029) and fewer developed arthritis (21% vs 39%, p = <0.001). Forty-three percent had a low risk, 49% a medium risk and 8% had a high to very high 10-year risk of cardiovascular mortality. Of those with a medium risk, 15% had a risk score above target according to the guidelines, qualifying them for drug treatment. Median QRISK3 score was 3.5% (1.6–9.7) with a median relative risk of 1.3 (1.0–1.8). Seven percent classified as high risk. SCORE and QRISK3 did not differ between preclinical arthritis and non-arthritis patients.

### Cardiovascular risk and ACPA status

In the group of preclinical arthritis patients, ACPA positive persons (n = 165) were younger (p = 0.006), had higher CRP levels (p = 0.003) and had lower TC (p = 0.015), LDL (p = 0.046) and ApoB (p = 0.012) compared to ACPA negative persons (n = 23; Table 2). SCORE and QRISK3 did not differ.

**Table 2:** Baseline characteristics of preclinical arthritis and non-arthritis patients, separated by ACPA status.

	Preclinical arthritis n = 188			Non-arthritis n = 367		
	ACPA + n = 165	ACPA–n = 23	p	ACPA +n = 183	ACPA–n = 184	p
Age (mean ±SD)	48.2 (11.2)	55.1 (9.5)	0.006	48.4 (11.8)	51.0 (11.7)	0.036
Sex, n (% female)	123 (75)	16 (70)	0.610	140 (77)	136 (74)	0.566
RF positive, n (%)	99 (60)	23 (100)	<0.001	70 (39)	184 (100)	<0.001
CRP (median, IQR)	2.0 (1.0–5.0)	1.0 (1.0–2.2)	0.003	2.0 (1.0–5.0)	2.0 (1.0–4.0)	0.295
Statin users, n (%)	16 (10)	3 (13)	0.618	26 (9)	13 (7)	0.551
Antihypertensive drug use, n (%)	25 (15)	2 (9)	0.539	23 (13)	33 (18)	0.153
History of CVD, n (%)	4 (2)	2 (9)	0.158	7 (4)	7 (4)	0.992
DM, n (%)	5 (3)	2 (9)	0.216	8 (5)	6 (3)	0.556
Current smoker, n (%)	52 (32)	4 (18)	0.227	55 (30)	46 (25)	0.264
SBP^1^ (mean ±SD)	129.1 (16.1)	142.7 (27.6)	0.139	127 (15.5)	128 (15.3)	0.944
Heart rate^1^ (mean ±SD)	66.1 (7.9)	72.3 (7.1)	0.099	65 (9.7)	62 (9.9)	0.169
BMI^2^ (mean ±SD)	26.2 (4.1)	24.9 (2.6)	0.447	26.0 (4.1)	26.2 (5.5)	0.774
TC^3^ (mean ±SD)	5.1 (1.0)	5.7 (1.1)	0.015	5.3 (1.1)	5.6 (1.2)	0.026
HDL^3^ (mean ±SD)	1.0 (0.4)	1.1 (0.4)	0.415	1.1 (0.4)	1.2 (0.2)	0.012
TC/HDL ratio^3^ (median, IQR)	5.4 (4.1–7.4)	5.6 (3.9–8.1)	0.886	5.2 (3.89–6.94)	4.9 (3.8–6.6)	0.392
LDL^3^ (mean ±SD)	3.5 (0.9)	3.9 (1.0)	0.046	3.6 (1.1)	3.7 (1.1)	0.180
Triglycerides^3^ (median ±IQR)	1.3 (1.0–1.8)	1.5 (1.2–2.4)	0.107	1.3 (0.9–1.9)	1.32 (0.9–2.0)	0.631
ApoA^3^ (mean ±SD)	1.6 (0.4)	1.6 (0.3)	0.892	1.7 (0.3)	1.7 (0.4)	0.094
ApoB^3^ (mean ±SD)	0.8 (0.2)	1.0 (0.2)	0.012	0.9 (0.27)	0.9 (0.2)	0.358
SCORE^4^ (median, IQR)	1.0 (0.0–2.0)	1.0 (0.0–2.0)	0.815	1.0 (0.0–2.0)	1.0 (0.0–2.0)	0.616
QRISK3^4^ score (median, IQR)	4.5 (1.6–15.6)	5.2 (2.2–9.1)	0.936	3.1 (1.1–9.6)	3.3 (1.8–9.8)	0.721
QRISK relative risk^4^ (median, IQR)	1.2 (0.9–1.9)	1.2 (0.9–1.7)	0.936	1.4 (1.0–2.0)	1.3 (1.0–1.8)	0.545

^1^ Measured in a subset; people with antihypertensive medication use were excluded from analyses. Included: n = 128 (preclinical arthritis: 26, non-arthritis: 102).

^2^ Height and weight was measured in a subset. Included: n = 160 (preclinical arthritis: 34, non-arthritis: 126).

^3^ Subjects using cholesterol lowering medication were excluded from analyses. Included: n = 507 (preclinical arthritis: 169, non-arthritis: 338).

^4^ For SCORE and QRISK3 calculation, subjects with a history of CVD were excluded. Included: n = 150 (preclinical arthritis: 31, non-arthritis: 119).

ACPA: anti-citrullinated protein antibodies, ApoA: apolipoprotein A, ApoB: apolipoprotein B, BMI: body mass index, CRP: C-reactive protein, CVD: cardiovascular disease, DM: diabetes mellitus, HDL: high density lipoprotein, LDL: low density lipoprotein, RF: rheumatoid factor, SBP: systolic blood pressure, TC: total cholesterol, TC/HDL ratio: total cholesterol / high density lipoprotein ratio.

In the group of non-arthritis patients, ACPA positive persons (n = 183) were younger (p = 0.036) and had lower TC (p = 0.026) and HDL (p = 0.012) levels than ACPA negative persons (n = 184; Table 2). SCORE and QRISK3 did not differ.

### Comparing seropositive at-risk individuals to seronegative controls

Seventy-one seronegative controls were matched in a 1:1 ratio for sex and age to seropositive arthralgia patients, those with the closest match were selected. The selected arthralgia patients did not differ from the unselected patients regarding sex, age and percentage of ACPA positivity, but less patients developed arthritis (21% vs 36%, p = 0.015). Compared to the seronegative controls, more people smoked (25% vs 7%, p = 0.005) and used antihypertensive drugs (24% vs 7%, p = 0.010) in the seropositive arthralgia group. Of all antihypertensive drugs used, 37% could increase serum lipid levels (hydrochlorothiazide, n = 6; sotalol, n = 1). Lipid profile and cardiovascular risk scores are shown in Table 3. In seropositive arthralgia patients, HDL was lower while TC, TC/HDL ratio, triglycerides, ApoB, SCORE and QRISK3 were overall higher compared to the seronegative controls.

**Table 3:** Cardiovascular risk of seropositive arthralgia patients and age and sex matched seronegative controls.

	Seropositive arthralgia patients	Seronegative controls	p
	N = 71 *	N = 71	
TC^1^ (mean ±SD)	5.4 (0.9)	4.9 (0.8)	<0.001
HDL^1^ (mean ±SD)	1.4 (0.5)	1.7 (0.4)	<0.001
TC/HDL ratio^1^ (median, IQR)	4.0 (3.4–5.0)	3.0 (2.4–3.5)	<0.001
LDL^1^ (mean ±SD)	3.2 (0.9)	2.9 (0.8)	0.098
Triglycerides^1^ (median ±IQR)	1.4 (1.0–2.1)	1.0 (0.7–1.5)	0.001
ApoA^2^ (mean ±SD)	1.6 (0.3)	1.6 (0.2)	0.736
ApoB^2^ (mean ±SD)	1.0 (0.2)	0.9 (0.2)	0.019
SCORE (median, IQR)	1.00 (0.00–2.00)	0.00 (0.00–1.00)	0.01
QRISK3 (median, IQR)	4.4 (2.0–9.8)	3.1 (1.5–6.2)	0.03
QRISK3 relative risk (median, IQR)	1.5 (1.1–1.9)	1.0 (0.8–1.2)	<0.001

* Of 71 patients, 15 (21%) developed arthritis

^1^ Subjects using cholesterol lowering medication were excluded from analyses. Included: n = 133 (seropositive patients: 67, seronegative controls: 66).

^2^ APOA and APOB were measured in a subset of seronegative controls (n = 37) and all seropositive patients (n = 67 excluding statin users).

ApoA: apolipoprotein A, ApoB: apolipoprotein B, HDL: high density lipoprotein, LDL: low density lipoprotein, TC: total cholesterol, TC/HDL ratio: total cholesterol / high density lipoprotein ratio.

## Discussion

In this large cohort study, preclinical arthritis patients had a higher heart rate and overall lower blood lipids compared to non-arthritis patients (i.e. at-risk patients who did not develop arthritis during follow up). Within the preclinical arthritis and the non-arthritis groups, ACPA positive individuals had lower lipid levels compared to ACPA negative individuals. The 10-year CVD risk scores did not differ between preclinical arthritis and non-arthritis patients and was not associated with ACPA status. Overall, persons at risk for RA showed a higher prevalence of traditional CVD risk factors compared to sex and age matched seronegative controls. These results confirm that early serum lipid changes commence prior to RA diagnosis and suggest that ACPAs might play a role.

The relatively lower lipid levels in preclinical arthritis patients are similar to lipid abnormalities seen in RA patients with untreated disease [20]. These results confirm our previous work in a substantially smaller seropositive arthralgia cohort [8]. Some other smaller studies [7, 21, 22] showed variation in the extent and direction of lipid changes, however, this also holds true for lipid level studies in early RA patients [1]. The mechanisms behind lipid changes in the preclinical and early phases of RA and their contribution to an increased CVD risk remain to be further unraveled. It is known that inflammation alters the function, composition and serum levels of lipids [23]. Data suggest that in RA patients, lipoprotein particle sizes and density, and their apolipoprotein cargo skew towards pro-atherogenic dyslipidemia which may contribute to the initiation and progression of atherosclerosis [24]. Additionally, inflammation may alter the anti-inflammatory and atheroprotective roles associated with HDL and turn this particle into pro-atherogenic instead of anti-atherogenic [25, 26]. These data imply that the traditional interpretation of lipid profiles for predicting CVD risk may be insufficient for RA patients with active disease and new strategies for lipid monitoring and treatment have been proposed [27]. In the preclinical phase of RA, low grade, subclinical inflammation might already cause similar lipid metabolism alterations [13, 28–31]. Interestingly, the difference in lipid profile between ACPA positive and ACPA negative individuals in both the preclinical arthritis and non-arthritis group might also indicate that ACPA itself plays a role in lipid metabolism changes and CVD risk. Thus far, the interplay between ACPAs and lipids has not been studied.

Remarkably, heart rate was higher in preclinical arthritis patients. These data confirm a study by Koopman et al. where in a small group of individuals at-risk for RA the mean heart rate was higher compared to healthy controls (68 bpm compared to 60 bpm) [32]. A higher heart rate is associated with increased cardiovascular morbidity and mortality [33, 34] and is also observed in RA patients [35–39]. An increase of 5 bpm translates into an approximately 20% increased CVD mortality risk [40]. This may be an additional factor, besides traditional CVD risk factors, contributing to the increased CVD risk at time of RA diagnosis.

The 10-year clinical cardiovascular risk scores were similar between preclinical arthritis and non-arthritis patients. Conversely, compared to seronegative controls, seropositive at-risk patients had a higher SCORE and QRISK3, reflecting an increased prevalence of traditional CVD risk factors. The difference in serum lipid levels between preclinical arthritis and non-arthritis patients, and between ACPA positive and ACPA negative patients were not reflected in the 10-year risk scores. It is important to note that these scores only incorporate traditional CVD risk factors, which only partly explain the increased CVD risk in RA patients [41, 42]. Therefore, these algorithms fail to identify a large part of high-risk patients in the RA population [43, 44]. Also in systemic lupus erythematosus (SLE) and psoriatic arthritis (PsA) patients, recent studies showed underperformance of SCORE and QRISK3 in identifying vascular ultrasound-based high CVD risk [45, 46]. This underperformance may also apply to the preclinical RA population. Therefore, other methods might be more appropriate to investigate early CVD development prior to RA such as measuring subclinical atherosclerosis by carotid intima-media thickness (IMT) [47, 48] or measuring arterial stiffness by pulse wave velocity (PWV) or magnetic resonance imaging (MRI) [49]. These techniques need further investigation in cohorts of persons at increased risk of RA and additionally, to fully understand the cardiovascular risk in this population, prospective studies monitoring cardiovascular events are needed. If we can identify measures that better capture early cardiovascular changes in seropositive patients at-risk for RA, we might be able to identify persons that would benefit from early primary prevention, enabling us to lower the increased CVD risk around the time of RA diagnosis.

Strengths of this study comprised the large, well-defined population of RF and/or ACPA positive arthralgia patients who were followed up to 5 years for arthritis development. Consequently, we had a large group of well-defined at-risk persons who did and did not develop arthritis. Furthermore, for additional analyses, we included a group of sex and age matched seronegative individuals that could serve as controls.

A limitation of this study is that information on medical history, comorbidities and medication use was collected by self-report which could have resulted in a slight underestimation of CVD burden in this cohort. However, data could generally be confirmed by hospital charts. Data on blood pressure and heart rate were collected in a subset of the cohort with slightly less ACPA positivity and arthritis development, possibly underestimating associations. Cardiovascular events were prospectively recorded from January 2018, and a longer data collection period is necessary to compare cardiovascular event data.

## Conclusions

Our results suggest that changes in serum lipid profile and heartrate commence prior to RA diagnosis and that ACPAs might be involved in the link between immune mechanisms, inflammation and lipid metabolism changes. These changes do not result in different 10-year CVD risk scores. Our findings encourage further research into CVD in persons at risk of RA. We suggest research into identifying more sensitive screening tools than clinical risk scores to display early cardiovascular changes that are not solely a result of traditional CVD risk factors, for example subclinical atherosclerosis and arterial stiffness as measured by carotid IMT, PWV and MRI. Additionally, the interplay between ACPAs, lipids and CVD is an important item for the research agenda. Finally, prospective collection of cardiovascular event data is needed.

## Supporting information

S1 Data (XLSX)

S2 Data (XLSX)
